# COVID-19 and Quarantine, a Catalyst for Ageism

**DOI:** 10.3389/fpubh.2021.589244

**Published:** 2021-04-12

**Authors:** Nathalie Barth, Jessica Guyot, Sarah Anne Fraser, Martine Lagacé, Stéphane Adam, Pauline Gouttefarde, Luc Goethals, Lauren Bechard, Bienvenu Bongue, Hervé Fundenberger, Thomas Célarier

**Affiliations:** ^1^SAINBIOSE Laboratory U1059 INSERM, University of Jean Monnet, Saint-Etienne, France; ^2^Chaire Santé des Ainés, University of Jean Monnet, Saint-Etienne, France; ^3^Gérontopole AURA, Saint-Etienne, France; ^4^Faculty of Health Sciences, Interdisciplinary School of Health Sciences, University of Ottawa, Ottawa, ON, Canada; ^5^The Department of Communication, Faculty of Arts, University of Ottawa, Ottawa, ON, Canada; ^6^Unité de Psychologie de la Sénescence, Université de Liège, Liège, Belgium; ^7^Department of Kinesiology, University of Waterloo, Waterloo, ON, Canada; ^8^Support and Education Technic Centre of Health Examination Centres (CETAF), Saint Etienne, France; ^9^Department of Clinical Gerontology, University Hospital of Saint-Etienne, Saint-Etienne, France

**Keywords:** COVID - 19, ageism, qualitative study, older adults, quarantine

## Abstract

In February 2021, France had more than 76,000 deaths due to COVID-19 and older adults were heavily affected. Most measures taken to reduce the impact of COVID-19 (quarantine, visit ban in nursing home, etc.) significantly influenced the lives of older adults. Yet they were rarely consulted about their implementation. Exclusion of and discrimination against older adults has been accentuated during the COVID-19 pandemic. While many articles discussing COVID-19 also mention ageism, few actually incorporate the perspectives and opinions of older adults. Our research aims to assess the ageism experienced by older adults during the COVID-19 pandemic. We conducted interviews with older adults (63–92 years, mean age = 76 years) in an urban area of France. Participants reported experiencing more ageism during the COVID-19 pandemic, including hostile and benevolent ageism from older adults' families. Despite reports of experiencing ageist attitudes and behaviors from others, however, older adults also identified positive signs of intergenerational solidarity during this COVID-19 crisis.

## Introduction

By January, 2021, after several months of the COVID-19 pandemic, France reported more than 68,000 deaths due to COVID-19. People aged 65 years and older in particular have been severely affected (1, 2). In France, 89% of hospital deaths were among people aged 65 and over (3). Deaths in nursing homes accounted for almost 40% of all deaths (4), while nursing home residents represented only 1% of the French population. These data only reflect the direct effects of COVID-19 on older adult mortality, and do not account for the indirect effects of COVID-19 on disruptions to prevention and management services for other health conditions (e.g., medical emergencies like stroke, delayed diagnoses of chronic diseases). Most of the measures taken to reduce the spread of COVID-19 (e.g., quarantines, visit bans in nursing homes) have influenced the lives of older adults, yet the voices and perspectives of older adults have been relatively invisible in media reporting, even when older adult health and quality of life are the topic of discussion.

Ageism refers to stereotyping, prejudice, and discrimination based on a person's age (5). Ageism is common in Western societies (6) and perceptions of older adults are often influenced by false beliefs and prejudices about senescence and dementia (7, 8). Ageism can be expressed in a hostile and explicit manner such as verbal abuse and neglect. It can also be manifested in a more subtle manner, such as patronizing language (e.g., or not allowing older adults to express their voice and choice on issues that directly concern them). Although the latter form, conceptualized as compassionate ageism or “benevolent ageism,” may reflect well-intentioned efforts to express kindness and concern for older adults, it stills reinforces negative stereotypes about the abilities and preferences of older adults, negates the diversity in older adults' abilities, preferences, and desires, and as such can negatively affect their self-esteem (9, 10).

Globally, ageism has been identified as the third most common basis for discrimination after racism and sexism (11). Public authorities explicitly and implicitly frame older adults as burdens on society and on families that must be supported, rather than a valuable segment of the population that can make meaningful contributions to public life and discourse (12). Dependence of older adults with higher care needs is perceived as an additional burden in individualistic societies where less cultural emphasis is placed on taking care of aging kin (13). However, this perspective does not reflect the majority of the older adult population, as dependent older adults represent a minority of the people aged 60 years and older (14).

Recent studies show that exclusion of and discrimination against people 65 years and older has been accentuated during the COVID-19 pandemic (15). This research describes discriminatory situations experienced by older people in the COVID-19 pandemic. An example of this from Italy is the release of ethical guidelines for the allocation of treatment in exceptional resource-limited situations by the Italian Society of Anesthesia, Analgesia, and Intensive Care (SIAARTI). The SIAARTI guidelines suggest that an age limit may need to be set for admission into intensive care (16). Several papers have shown the importance of this subject and its consideration by scientists (17–19). The European Union, France, and other countries have talked about the end of quarantine by age category and extended for older adults (20). While these measures are meant to be protective for an age group at greater risk of death and serious health complications from COVID-19, the application of these measures to a specific age group without consultation of the affected group or consideration of the negative impacts of these protective measures on them can be considered ageist. Measures to reduce the impacts of COVID-19 significantly affect older adults, who paradoxically have little voice in these matters (21). While many articles mention ageism, few have examined the representations and opinions of those older adults (22, 23).

## Objectives

The study aimed to assess the ageism experienced by older adults living in two “departments” (similar to counties) in the center of France during the COVID-19 pandemic in April-May 2020.

## Method

### Ethics

This study received review by and approval from the Ethics Committee of the Saint-Etienne University Hospital Center (IRBN452020/CHUSTE).

### Methods

To survey the perspectives and opinions of older adults, semi-structured interviews were conducted with older adults in April and May of 2020 during the “first wave” of the COVID-19 pandemic in France. With older adults largely under-represented in the media during this early phase of the COVID-19 pandemic, the researchers believed it was important to use a methodology that captured the voices of older adults. Telephone interviews were conducted with older adults aged 65 years and older living at home in Auvergne-Rhône-Alpes, France. Auvergne Rhône-Alpes is an area in the center of France with large conurbations that polarize vast urbanized territories. Older adult participants were recruited through local older people's associations and community and social action centers. We contacted the persons in charge of these organizations by telephone or e-mail so that they could distribute our request. The participants were then drawn at random from among those who had agreed to participate. Interviews were conducted with until theoretical saturation was achieved, and additional interviews did not elicit any novel themes (24).

The aim of these interviews was to collect the experiences of older adults living at home during the COVID-19 pandemic, including experiences of ageism. Interview questions covered four categories: (1) experiences of ageism in quarantine, (2) relationships with family and friends, (3) reported feelings of discrimination, (4) experiences at the end of the quarantine. Older adults were asked open-ended questions about the quarantine, if they felt discriminated against, and whether or not they understood the concept of social distancing. The interviews were audio-recorded, transcribed, and analyzed using a thematic analysis method (25) and the NVivo qualitative analysis software platform (QSR International). The initial data corpus was composed of interview transcripts. Researchers completed initial open coding stage to identify key thematic elements in the transcripts. This process of coding-on was completed for the entire data corpus (26). Horizontal thematic analysis was then conducted to identify recurring themes and understand each individual's responses to a given theme. Following this, vertical analysis was completed by the researchers to observe possible links between the COVID-19 pandemic, feelings of stigmatization experienced by older adults, and discrimination experienced by older adults, and reflect each individual's responses for all the identified themes (27). The interviews were conducted in French, transcribed verbatim, then translated into English. The translation was carried out by bilingual research team members (S.F., M.L.).

## Results

We conducted telephone interviews with 20 older adults (63–92 years, mean age = 76 years) living at home. Interviews lasted 28 min on average (18–37 min). We interviewed eight men and 12 women, half of whom lived alone and the other half with a partner. Most older adults interviewed lived independently in urban areas. Table 1 reports the participant characteristics.

**Table 1:** Characteristics of respondents.

**N^**°**^**	Participants^*^	**Gender (12 women, 8 men)**	**Age (mean age = 76 years)**	**Current living situation**	**Dwelling location**
1	Michèle	F	70–75	Lives alone	Urban
2	Jeannine	F	60–65	Lives alone	Urban
3	Sylvie	F	+90	Lives alone	Urban
4	Christine	F	65–70	Lives with a partner	Urban
5	Gérard	M	75–80	Lives with a partner	Urban
6	Gisèle	F	+90	Lives alone	Urban
7	Pierre	M	80–85	Lives with a partner	Rural
8	Martine	F	65–70	Lives alone	Urban
9	Carine	F	65–70	Lives alone	Urban
10	Nicole	F	65–70	Lives alone	Urban
11	Roger	M	80–85	Lives with a partner	Urban
12	Jean Pierre	M	80–85	Lives with a partner	Urban
13	Marc	M	80–85	Lives with a partner	Urban
14	Philippe	M	80–85	Lives with a partner	Urban
15	Eric	M	65–70	Lives with a partner	Rural
16	Annie	F	65–70	Lives with a partner	Rural
17	Marie	F	85–90	Lives alone	Urban
18	Lucie	F	+90	Lives with a partner	Urban
19	François	M	65–70	Lives with a partner	Urban
20	Brigitte	F	70–75	Lives alone	Rural

* *All participant names are pseudonyms to protect the privacy and confidentiality of participants*.

### Perspectives of Older Adults

#### Step Back to Make Way for the Younger Ones

Two of 20 older adults agreed with this prioritization: “*I don't want to take the place of others, I'm not afraid for myself, considering my age” (Marie*^1^*)*. While most of the participants reported being against this type of prioritization, many were resigned to it, suggesting the presence of internalized ageism: “*for us, it's not the worst, we're certainly not too far from the end” (Lucie)*.

Due to the lack of intensive care equipment, some countries (e.g., Italy) have formally prioritized treatment of younger adults in resource-limited intensive care settings over older adults due to a higher likelihood of recovery for younger patients (16). We observed words like “*sacrifice*” used in the media reporting to describe this prioritization were similarly used by older adults interviewed in this study, but mostly for older people over 80 years old.

#### Ageism Within Families

Adult children were described by older adult participants as developing strategies to keep their parents from going out, such as running everyday errands for their older parents. Adult children were perceived by older adult participants as discouraging them from leaving the house and insisting on doing the shopping for them instead: “*Since I came home on Friday the 13th of March, I haven't gone through the door of the apartment, not even to buy bread” (Eric)*. Under the guise of “protection,” there is an inverted role of authority. Older adults described feeling infantilized by their loved ones, but excused their adult childrens' behaviors quite readily (eight out of 20 participants). “*[Our] children wrap us in bubble wrap […] but they worry too much, we do it [follow their restriction] to reassure them.” (Annie)*. Older adult participants thought from the perspectives of adult children to justify their protective behaviors, remembering when they had done the same thing for their aging parents, reflecting an element of ageism within family dynamics: “*When my son says,” No, but Daddy let me. “If I were in his place, I would have said yes! It's appealing, but at the same time it's pleasing, he doesn't do it at all out of discrimination” (François)*.

Older adults saw their loss of independence in everyday activities as a “sacrifice” made in concession to appease their loved ones. This constitutes a form of benevolent ageism, consisting of positive attitudes such as helping older people based on negative representations of aging, paternalism, and infantilizing attitudes.

#### Is the End of Quarantine Conducive to Intergenerational Tensions?

In France, residents were expected to adhere to a strict period of quarantine from March 17 to May 11, 2020. During this time, movement outside the home was restricted to essential activities. The older adults interviewed in this study reported having adjusted to their new way of living under quarantine, but feared the end of quarantine (10/20 participants). The end of the quarantine scared these participants because they feared younger people would not respect the public health guidelines for reducing COVID-19 transmission after the quarantine was lifted: “*I think that the older adults are quite capable of applying restrictive measures to be careful not to go out incorrectly [not following the rules]. It seems to me that when we are younger, we believe that nothing can happen to us and we are often less careful.” (Martine)*. Several participants believed the behaviors of younger adults could have repercussions on restrictions faced by older adults during the end of quarantine.

#### A Discriminatory “Older Adults” Categorization

On April 15, 2020 before the French Senate, the President of the Scientific Council for COVID-19 called for an extension of the confinement for people over 65 years of age^2^. Following that, government announcements calling for the extension of quarantine for this age group increased. Almost all older adult participants interviewed in our study (18/20) felt discriminated against by these announcements. Our younger respondents (under 70 years old), were outraged by this form of discrimination: “*When they said that the older adults were not allowed to go out. we're not pests! We're old enough to know what to do!” (Christine)*. This outrage can also be explained by the fact that most younger participants did not view themselves as “older adults” (7/8 participants under 70 years old): “*When they started talking about the end of quarantine, I said to myself” ah shit, I'm in it! “I wanted to tell myself that I wasn't concerned.” (François); “I don't feel old. I ride my bike and last summer, my daughter called me for her moving day, to help her carry things!” (Eric)*. Based on these and similar statements made by younger participants, consideration of “older adults” as one homogenous group did not correspond to how they felt they should be treated in reality: “*In my head, I don't feel old” (François)*.

#### The Isolation Is Much Harder to Bear

Most older adult participants (18/20) found the most difficult part of the COVID-19 pandemic was not the discrimination based on their age, but the isolation they experienced. What they missed the most was seeing their children and grandchildren. The older adult participants longed for shared intergenerational moments (19/20): “*Jérôme, my son, I told him as soon as you can get out, your first visit will be for me […] Vanessa, my granddaughter, she lived with me during her studies, I miss her” (Marie); “For us, the most difficult thing is that we don't see our children and grandchildren anymore. We deal with the rest [of the pandemic].” (François)*.

## Discussion

The objective of this study was to assess the ageism experienced by older adults in France during the first wave of the COVID-19 pandemic in 2020. Interviews with older adults in this study revealed the presence of ageism in the policy recommendations developed in response to the COVID-19 pandemic. A brief review of media using the Europresse database and including all articles published in national and international newspapers confirms this trend. This review was conducted to compare the number of articles on ageism published prior to the COVID-19 pandemic (January 2019 to February 2020) and those following the onset of the COVID-19 pandemic (February 2020 to October 2020). We found several articles that discussed the feelings of older adults during this period of COVID-19, with many reporting incidents of discrimination against older adults. The results show an increase in the number of newspaper articles during the COVID-19 period discussing ageism compared to the same period during the previous year. This increase in references to ageism is also tied to greater publication of aging-related media in general (Table 2).

**Table 2:** Press review.

**Keywords**	**Press**	**January 2019-February 2020 (13 month)**	**February 2020- October 2020 (8 month)**	Multiplier factor between the 2 periods^*^
French research: ≪ ageism ≫ and ≪ older people ≫	French press	7	8	X 1.9
French research: ≪ Discriminations ≫ and ≪ older people ≫	French press	10	16	X 2.5
≪ Ageism ≫	International press	749	2,627	X 5.7
≪ Ageism ≫ and ≪ older ≫	International press	68	110	X 2.6
≪ Ageism ≫ and ≪ elderly ≫	International press	102	310	X 4.9
≪ Ageism ≫ and ≪ discrimination ≫ and ≪ elderly ≫	International press	38	113	X 4.8
≪ Older ≫	International press	183,169	382,570	X 3.4
≪ Older people ≫	International press	102,045	254,632	X 4.1
≪ Elderly ≫	International press	58,855	153,823	X 4.2

* *(“period 2”/8 month)/(“period 1”/13 month)*.

Rapid dissemination of media *via* social networks led to a surge in discriminatory and ageist media early in the pandemic (28). For example, a 90-year old woman who died of COVID-19 in Belgium became a Facebook “hero” after saying: “*save it* [the ventilator] *for the youngest”* (29). New research is investigating the negative impact of ageist stereotypes on older adults within families (30). With these ageist behaviors, older adults feel disempowered to take an active role in their health and deprived of their status as “capable” persons, calling into question the self-determination of older adults (31). Self-determination of older adults is a World Health Organization priority, as it enables improved health outcomes for older adults. Developing conditions that empowers individuals to take an active role in improving their health status supports improved population health (32).

Discrimination against older adults after lifting quarantine measures in France seems to have also increased. Several countries, including Israel and the United Kingdom, have chosen to impose stricter quarantine measures on older adult citizens (70 years and older) compared to younger age groups (29). These measures are often justified by higher mortality rates among older persons who have additional risk factors increasing their likelihood of more severe COVID-19 infection and fatality (33). However, these measures perpetuate the attitude that “physiological” health is the most important factor in overall health and minimize the importance of other aspects of health and well-being (e.g., mental, emotional) that have been negatively impacted by quarantine and social distancing measures. Disease severity is often most closely associated with the severity of its physiological symptoms as this provides a visible, tangible indicators of the lethality of a disease, with the psychosocial implications of the disease considered after the fact. Quarantine and the isolation of the older adults, especially in institutional settings like nursing homes, can also lead to “syndrôme de glissement” (“failure-to-thrive syndrome”). This French geriatrics concept is a psychopathological mechanism close to depression and is often linked to the unstimulating environments (34). Forbidding people to go outside based only on chronological age, not the state of one's health and resilience, can be interpreted as a form of ageism. Quarantine and social isolation were established to limit COVID-19 mortality in older adults, but do not come without their own risks (35, 36). Social isolation is a major health risk for older adults. One study from Sweden found people with few social contacts had a mortality rate 3.7 times higher than people with many social contacts (37).

While other countries have chosen to follow criteria informed by additional factors beyond one's chronological age, the vaccination campaign in France continues to use classification by age group in planning and policy decisions (38). The most telling case of this is in the policy around caregiver vaccinations, where the first choice was to vaccinate people aged 50 and over. Age-associated risk of death and severe morbidity from COVID-19 could suggest some conditions under which age-focused action or prioritization (such as vaccination). But in terms of exposure, the risk is the same for all caregivers regardless of their age. Age categorization accentuates differences between groups and the homogenisation of intra-group characteristics. It facilitates the formation of stereotypes, prejudices and discrimination against the group (39). This type of policy has already been questioned by the French Academy of Medicine. Older adults should not be considered a homogeneous group (40).

The selective extension of quarantine for older adults in France will likely worsen the impact of social isolation on their health and well-being (41). This was the greatest concern of the older adults we interviewed. It is important to refrain from broad age-based categorizations that are not based on best evidence in the COVID-19 response and expose older adults to greater social isolation than is necessary (16, 42). Finally, more research examining the promotion of intergenerational supports during the COVID-19 pandemic is needed to reduce the focus of media and research on the negative, ageist effects of the COVID-19 pandemic.

## Conclusion

During the first wave of the COVID-19 pandemic in France, there was an increase in ageist narratives in the media. Media, policy briefs, and commentaries during the early phase of the COVID-19 pandemic and age-specific quarantine measures suggest that ageist attitudes exist and are being shared through public forums. Age-based discrimination and ageist attitudes were experienced by older people in France during the first period of quarantine in 2020. This discrimination was present in formal measures and public narratives as well as more informally within family networks. For older adults in this study, concerns about the impacts of social isolation were more troubling than experiences of ageism in the COVID-19 pandemic. Older adult participants in this study were less concerned about dying from COVID-19 and policies put in place related to the care of the elderly than they were about the effects of social isolation. In future quarantine periods in France during subsequent waves of the COVID-19 pandemic, research should continue to examine the evolution of ageism in responding to the COVID-19 pandemic, policy recommendations and whether older adults continue to have similar experiences of age-based discriminations as reported during the first quarantine period.

## Data Availability Statement

The original contributions presented in the study are included in the article/supplementary material, further inquiries can be directed to the corresponding author/s.

## Ethics Statement

The studies involving human participants were reviewed and approved by the ethics committee of the Saint-Etienne University Hospital Center IRBN452020/CHUSTE. The patients/participants provided their written informed consent to participate in this study.

## Author Contributions

All authors listed have made a substantial, direct and intellectual contribution to the work, and approved it for publication.

## Conflict of Interest

The authors declare that the research was conducted in the absence of any commercial or financial relationships that could be construed as a potential conflict of interest.

## Footnotes

^1^For the sake of anonymity all names have been changed.

^2^National Assembly Commission of April 15, 2020 - Jean François Delfraissy - President of the Scientific Council COVID-19.
